# Open questions in human lung organoid research

**DOI:** 10.3389/fphar.2022.1083017

**Published:** 2023-01-13

**Authors:** Tessa Hughes, Krijn K. Dijkstra, Emma L. Rawlins, Robert E. Hynds

**Affiliations:** ^1^ Wellcome Trust/CRUK Gurdon Institute and Department Physiology, Development and Neuroscience, University of Cambridge, Cambridge, United Kingdom; ^2^ Department of Molecular Oncology and Immunology, The Netherlands Cancer Institute, Amsterdam, Netherlands; ^3^ Cancer Evolution and Genome Instability Laboratory, The Francis Crick Institute, London, United Kingdom; ^4^ Epithelial Cell Biology in ENT Research (EpiCENTR) Group, Developmental Biology and Cancer Department, Great Ormond Street UCL Institute of Child Health, University College London, London, United Kingdom; ^5^ CRUK Lung Cancer Centre of Excellence, UCL Cancer Institute, University College London, London, United Kingdom

**Keywords:** lung stem cells, respiratory biology, 3D cell culture, in vitro models, epithelial cells

## Abstract

Organoids have become a prominent model system in pulmonary research. The ability to establish organoid cultures directly from patient tissue has expanded the repertoire of physiologically relevant preclinical model systems. In addition to their derivation from adult lung stem/progenitor cells, lung organoids can be derived from fetal tissue or induced pluripotent stem cells to fill a critical gap in modelling pulmonary development *in vitro*. Recent years have seen important progress in the characterisation and refinement of organoid culture systems. Here, we address several open questions in the field, including how closely organoids recapitulate the tissue of origin, how well organoids recapitulate patient cohorts, and how well organoids capture diversity within a patient. We advocate deeper characterisation of models using single cell technologies, generation of more diverse organoid biobanks and further standardisation of culture media.

## 1 Introduction

Organoid cultures are *in vitro* models derived from stem/progenitor cells, and involve the generation of a heterocellular structure that is reminiscent of the tissue of origin in a three-dimensional cell culture environment (Barkauskas et al., 2017; Liberti and Morrisey, 2021; Sen et al., 2022). In the human respiratory system, nasal (Liu et al., 2020; Rodenburg et al., 2022), tracheobronchial (Rock et al., 2009; Sachs et al., 2019), small airway (Basil et al., 2022) and alveolar (Katsura et al., 2020; Salahudeen et al., 2020; Youk et al., 2020) epithelial organoid models have all been generated from post-natal tissue-resident stem cells. Additionally, organoid cultures derived from developing lung epithelia have been described (Nikolić et al., 2017; Miller et al., 2018), and the stepwise differentiation of pluripotent stem cells has been used to derive mature lung organoids and those resembling developmental intermediates (Jacob et al., 2017; Hawkins et al., 2021; Hein et al., 2022). In addition to providing a platform to study lung stem cell biology, organoids present a platform to investigate respiratory diseases, including developmental disorders such as bronchopulmonary dysplasia (Riccetti et al., 2022), genetic disorders such as cystic fibrosis (Sachs et al., 2019) and ciliary dyskinesias (van der Vaart et al., 2021a), chronic respiratory diseases such as chronic obstructive pulmonary disease (COPD) (Ng-Blichfeldt et al., 2018) and lung cancers (Sachs et al., 2019).

Recent, comprehensive reviews are available on advances in methods to derive lung organoids, and the potential uses of organoids in basic research and translational medicine (Evans and Lee, 2020; van der Vaart et al., 2021b; Liberti and Morrisey, 2021; Sen et al., 2022). While lung organoids can also be derived from mouse lung epithelia, offering an opportunity to address some research questions *in vitro* and reduce the use of animals used in model species research, we will focus on pertinent open questions within the human lung organoid field with relevance across developmental, post-natal stem cell and cancer studies.

## 2 How closely do lung organoids recapitulate their tissue of origin?

To increase the likelihood of relevant findings, it is crucial that organoids mimic the tissue of interest as closely as possible. To this end, the development of multiple organoids to capture the diversity of cells within developing lungs, adult homeostatic lungs and tumours has been ongoing. It is important to note that in developmental studies, organoids are typically derived from progenitor cells that are capable of differentiation into different tissue types (e.g., airway and alveolar), while adult lung organoids are typically region-specific with less plasticity.

Developmental studies currently tend to favour the use of induced pluripotent stem cells (iPSCs) due to issues of scalability, availability and the fact that iPSCs represent a better-defined starting population than those available from foetal tissue. Customised media have been developed to expand specific cellular subpopulations from iPSCs; in the airways, basal cells and secretory progenitor cells have all been induced from iPSCs (McCauley et al., 2017; McCauley et al., 2018; Hawkins et al., 2021) and, when cultured at an air-liquid interface, goblet cells and ciliated cells have also been generated from iPSCs (Firth et al., 2014; McCauley et al., 2017). For alveolar modelling, iPSCs and human embryonic stem cells (hESCs) have been used to derive AT2 cells (Jacob et al., 2017; 2019; Tamò et al., 2018; Hurley et al., 2020), which can exhibit relevant morphological characteristics, such as lamellar bodies and microvilli. However, although some iPSC-derived AT2 organoids are stable in long-term culture, there are reported stability issues with cells reverting to non-lung fates.

At the histological level, airway and lung cancer organoids can resemble their *in vivo* tissue of origin remarkably well, despite the absence of stroma *in vitro* (Kim et al., 2019; Sachs et al., 2019; Li et al., 2020). Airway organoids contain all of the major airway epithelial cell types, with basal cells, mucosecretory cells and ciliated cells present (Rock et al., 2009; Sachs et al., 2019), although the consistency with which they contain rare populations such as neuroendocrine cells, ionocytes and tuft cells is unclear. Removing extracellular matrix cues can generate organoids with cilia on their outer surface, which has clear advantages for studies of motile cilia (Wijesekara et al., 2022). Media composition appears to be vital in the selection of airway vs. alveolar cells from adult lung tissue, as studies using distal lung resection tissue for organoid generation have led to airway organoid formation but an absence of alveolar organoids (Sachs et al., 2019), in contrast to earlier mouse lung organoid studies in which both organoid types were observed within the same cultures (Lee et al., 2014). The resemblance of alveolar organoids to the alveolus is more limited given the dependence of this tissue on precise architecture. Alveolar organoids typically consist of proliferative AT2-like cells but lack AT1-like cells, though cells resembling AT1 cells can be induced by 2D culture (Youk et al., 2020), suspension culture (Salahudeen et al., 2020) or using inductive culture media (Katsura et al., 2020; Konishi et al., 2022).

Although progenitor differentiation to mature adult cell types is a common goal, the lack of complete lineage characterisation hinders our ability to fully distinguish these differentiated cell types. At present, many iPSC studies accomplish progenitor specification but are not able to fully substantiate claims of mature cells. Marker protein expression remains the most common means to identify differentiated cell types within organoids, which is not in itself sufficient to demonstrate full maturity or functionality. Additional confusion arises when mouse markers are extrapolated to human lung cell types as, particularly in the developing lung, numerous distinct cell types can share marker expression (Miller et al., 2020; He et al., 2022) and marker expression can be transient (McCauley et al., 2018). To overcome this, recent studies have combined marker expression data with spatial information, transcriptomic analysis and organelle characterisation (Dye et al., 2015; Miller et al., 2019; 2020; Sachs et al., 2019; Hurley et al., 2020; Hein et al., 2022). The derivation of organoids that capture the intermediary stages of lung development has also been explored. Bud tip progenitors can be derived from iPSCs, which in turn have the potential to differentiate into all lung epithelial cell types (Hein et al., 2022). iPSCs were also used to culture a progenitor population enriched in basal cells that were reminiscent of those from foetal lungs (Ngan et al., 2021).

The increase in single-cell and bulk RNA sequencing studies of human lung is allowing greater benchmarking of organoid systems and comparisons of iPSC and embryonic tissue-derived cells (Ngan et al., 2021; Alysandratos et al., 2022; He et al., 2022; Murthy et al., 2022). Although cell culture often produces less robust and less mature differentiated cells, iPSC-derived organoids frequently produce more immature cell types than hESC-derived organoids, citing the need for additional validation in these studies, such as through lineage tracing or trajectory analysis (Hurley et al., 2020; Hawkins et al., 2021). In protocols that highlight stepwise differentiation and intermediary stages, the order of differentiation should be carefully analysed, such as by visualising differentiating structures, ensuring proximal-distal cell patterning has been conserved, and/or by matching with corresponding *in vivo* sequencing data. This level of detail allows for a sequence of key developmental milestones and transitional states to be uncovered, and potentially better recapitulation of human foetal lung development (He et al., 2022). A major limitation across lung organoid studies is the lack of direct comparison to the *in vivo* tissue, and as yet, the field has not created a standardised protocol for definitively assessing cell maturity and identity. Additionally, there is a large amount of variation between iPSC lines, with some unable to generate mature lung cells.

In contrast to developmental and adult lung organoid studies, the resemblance of cancer organoids to their tumours of origin has been most extensively studied at the genomic level. There is a relatively high concordance (± 80%) of single nucleotide variants (SNVs) and copy number alterations (CNAs). Although whole exome sequencing has been performed in some studies (Kim et al., 2019; 2021; Shi et al., 2020; Hu et al., 2021), others have investigated a limited set of cancer driver genes (Sachs et al., 2019; Chen et al., 2020; Li et al., 2020). Since targeted approaches tend to target clonal mutations (those that are found in every cancer cell), they identify mutations that are less susceptible to loss during organoid culture. Whether lung cancer organoids recapitulate the full spectrum of (subclonal) cancer mutations is not yet clear; while on average the genetic concordance between organoids and tumour tissue is high, for some samples the concordance is considerably lower (Kim et al., 2019; Shi et al., 2020), which most likely reflects the selective outgrowth of minor tumour subclones in those organoid cultures.

The extent to which lung cancer organoids diverge from tumour tissues at the phenotypic level is much less clear. Since organoid culture systems were originally developed to expand epithelial stem cells (Sato et al., 2009), it is unclear if lung cancer organoids can reflect tumour cells with different degrees of differentiation. Organoids established from distinct histological subtypes retain characteristic features, such as a cystic morphology for organoids from acinar adenocarcinoma, and a solid morphology for organoids from the solid subtype (Li et al., 2020). This suggests that tumour histology is at least in part shaped by tumour-intrinsic factors and independent of the tumour microenvironment (TME). The few reports of transcriptomic comparisons of lung cancer organoids and primary tissue are limited to overall concordance rates, which is (as expected) lower than genomic similarity, partially due to the absence of TME pathways (Shi et al., 2020; Hu et al., 2021). The absence of the TME might have profound effects on organoid phenotype and drug responses, as has been shown for pancreatic adenocarcinoma organoids (Raghavan et al., 2021). A more thorough transcriptomic and proteomic comparison of organoids and tumour tissues is needed to identify where divergence occurs. While organoids can converge upon culture specific transcriptional states, these can be modified by adding “missing” paracrine signals from the TME to the media (Raghavan et al., 2021). Alternatively, the TME can be partly reconstituted by adding back cell types of interest, such as lymphocytes or fibroblasts (reviewed in (Fiorini et al., 2020)).

The majority of lung organoid cultures are currently performed as mono (epithelial) cultures, although investigations of epithelial-fibroblast (Tan et al., 2019) or epithelial-macrophage (Iakobachvili et al., 2022) interactions have been possible. Epithelial-mesenchymal organoids have been developed from both embryonic cell lines and iPSCs, which contained basal cells, immature ciliated cells, smooth muscle and myofibroblasts (Dye et al., 2015). Additionally, iPSC differentiation generated organoids that contained bronchi-like structures surrounded by mesenchyme, and cells expressing alveolar markers (Miller et al., 2019). Multi-organ systems have also begun to be developed; iPSCs can be differentiated into cardiac and lung epithelial lineages to establish cardio-pulmonary micro-tissues (Ng et al., 2022).

While we advocate a better phenotypic characterisation of lung organoids to investigate their resemblance to lung tissue, it must be emphasised that organoids will always remain a reductionist model. The generalisation or translational relevance of results obtained in this culture system is best validated using orthogonal model systems (e.g., mouse models, 2D primary cultures, precision-cut lung slices) or datasets (including publicly available omics datasets). Moreover, it is not possible to reproduce the complete *in vivo* complexity of epithelial-stromal or epithelial-immune interactions, nor systemic variables such as nutrient availability, mechanical forces and circadian rhythms.

## 3 How well do organoid cultures represent patient cohorts?

A second important aspect of organoid modelling is the extent to which it is possible to capture the biology of the target population. Primary human bronchial epithelial cell cultures can show wide inter-patient variability in differentiation potential and response to stimulation, and so studying sufficient numbers of patient cultures is crucial, particularly when studying phenomena that are likely to vary with biological characteristics of the donor, such as age and/or sex (Peretz et al., 2016; Maughan et al., 2022). At present, organoid studies typically investigate cells isolated from small numbers of human donors and these cultures are rarely common between investigations from independent laboratories, due to limitations around access to human material, restrictions imposed by ethical approvals, and administrative and practical difficulties in sharing tissue or cultures within and between countries.

Organoids have been derived from patients across a wide range of respiratory diseases, including pulmonary fibrosis (Surolia et al., 2019), primary ciliary dyskinesia (van der Vaart et al., 2021a) and cystic fibrosis (Sachs et al., 2019). An extensive organoid collection from 664 cystic fibrosis patients has been reported (Geurts et al., 2020) meaning that models are now available for a wide range of causative mutations; however, these are rectal rather than lung organoids, since airway organoid forskolin-induced swelling is highly variable and limited to well-differentiated organoids. However, in chronic respiratory diseases the use of patient-derived organoids has been limited, likely due to a combination of the difficulty in obtaining material from patients with COPD and pulmonary fibrosis, the invasive nature of procedures to obtain samples, and the intrinsic lower potential of cells from these patients to generate organoid cultures (Ghosh et al., 2018). The extent to which organoid collections capture the diversity within patient cohorts will be disease-specific and depend on factors such as the opportunity to obtain research biopsies (clinically, but also geographically), the stage at which disease is most commonly diagnosed, and/or the diversity of disease phenotypes. iPSC technology might help to improve the range of chronic respiratory disease organoid models available, although pluripotent cell derived-organoids can have immature phenotypes, resembling fetal, rather than adult, transcriptional profiles. Indeed, a recent study of non-alcoholic fatty liver disease pooled iPSC-derived progenitors cells from 24 genotyped donors and derived mixed donor organoid cultures, before inducing a disease-relevant phenotype to investigate phenotype-genotype interactions (Kimura et al., 2022), suggesting that in the future lung organoid models may also have a role in identifying risk factors in addition to studying disease pathogenesis.

To date, lung cancer research has benefited most from organoid-based disease modelling. As many hundreds of organoid lines have been developed by multiple laboratories worldwide, it is possible to assess the extent to which they reflect non-small cell lung cancer (NSCLC) patient cohorts. Surprisingly, organoid establishment rates are similar for lung squamous cell carcinomas or adenocarcinomas (Kim et al., 2019; Shi et al., 2020; Hu et al., 2021), despite their distinct cells of origin, divergent driver gene landscapes, and the use of one medium composition across histological subtypes. Organoids for diverse histological subtypes (e.g., acinar, lepidic, or solid adenocarcinomas) (Kim et al., 2019; Li et al., 2020) have been reported, although a systematic comparison of organoid establishment across subtypes is lacking. It is likely that current approaches are biased towards establishing organoids from certain tumour phenotypes and/or genotypes. Moreover, each of these studies differ in patient cohorts, the tissue digestion protocol used, medium formulation, and criteria of what is considered a successful culture, making it challenging to determine important contributors to successful cultures and resulting in organoid establishment rates of between 15% and 80% (Ma et al., 2022). The field would benefit from standardisation, including systematic comparisons of different protocols. Given the heterogeneity of lung cancer there may not be a “one size fits” medium formulation, and the choice of medium could be guided by driver gene status or features of the tumour TME (Fujii et al., 2016). Larger studies should explore any transcriptional or genomic features that are associated with culture success. Given the heterogeneity of lung cancer, this will likely be best achieved through large-scale collaborative efforts.

To date, most studies have generated lung cancer organoids from surgical resections of primary tumours. To our knowledge, no organoid models have been generated from pre-invasive disease, but pre-cancer organoids would be particularly valuable to study events in early tumourigenesis. Since most lung cancer patients die from metastatic disease, representing these in organoid collections would be valuable. One study established organoids from malignant effusions from patients with advanced lung cancer with a high success rate (Kim et al., 2021), suggesting that establishment rates from metastatic disease could be higher than for early stage disease. Organoid establishment from extrapulmonary metastases can also avoid contamination with normal airway organoids (Sachs et al., 2019; Dijkstra et al., 2020).

An alternative approach to deriving organoids from lung disease patient cohorts, is to use the available model systems to investigate specific hypotheses related to the disease process. In this regard, pluripotent cell-derived organoids have proved useful pulmonary fibrosis models. Introducing Hermansky-Pudlak syndrome (HPS) mutations associated with interstitial pneumonia using CRISPR-Cas9 promotes fibrotic changes in ES-derived lung organoids (Strikoudis et al., 2019) or iPSCs from patients (Korogi et al., 2019), while treatment of iPSC-derived AT2 organoids with bleomycin also promotes fibrotic changes (Suezawa et al., 2021). Moreover, early disease models can be generated by recapitulating aspects of disease development in lung organoids. For example, insights into pulmonary fibrosis have arisen from treating cells with TGFb (Ng-Blichfeldt et al., 2019). These approaches, akin to the sequential introduction of cancer driver mutations into normal organoid cultures to study early events in tumourigenesis (Dost et al., 2020), have the potential to reveal new insights compared to organoids from established disease.

## 4 To what extent does an organoid culture capture diversity within a patient?

Intra-patient heterogeneity likely represents an understudied source of variation in lung organoid research. At present, authors typically report the diagnosis of the patient from whom organoids were derived and the tissue of origin. However, airway epithelial cell phenotype is known to vary in the proximal-distal axis within normal lungs with cells from the upper airways having distinct transcriptomic profiles (Deprez et al., 2020; Hou et al., 2020), innate immune defences (Mihaylova et al., 2018), and susceptibility to infection (Hou et al., 2020), compared to those from the lower airways. As an example, organoid origins (proximal versus distal, as well as pluripotent versus adult) might partially explain the divergent cell types that have been seen to be infected with SARS-CoV-2 in lung organoid studies (Han et al., 2022). Moreover, patients with chronic respiratory diseases might display additional heterogeneity, particularly as IPF distal airway epithelial cells respond differently in cell culture to proximal epithelial cells from the same patient (Stancil et al., 2021). Since pulmonary fibrosis is characterised by upper lobe emphysema and lower lobe fibrosis, the environment that cells experience even within the same lung varies and might influence their subsequent behaviour. As such, the precise location of a biopsy is likely to be consequential for experimental reproducibility.

Lung cancers are genetically and phenotypically heterogeneous, but the extent to which this is maintained in lung cancer organoid cultures is so far poorly characterised. Given the variable establishment rates of lung cancer organoids, bottlenecks are expected that restrict heterogeneity *in vitro*. Indeed, one study reported a low correlation of variant allele frequencies (VAFs) between organoids and original tumours in approximately half of the samples analysed (Kim et al., 2019). Evidence for selective subclonal outgrowth has also been observed in bladder (Lee et al., 2018) and colorectal cancer organoids (van de Wetering et al., 2015). This could be due to sampling bias, selective clonal outgrowth, or tumour evolution driven by ongoing genetic instability. While the genetic landscape of organoids is relatively stable during long-term culture (Kim et al., 2019), there is evidence for ongoing genetic instability and clonal selection from single cell sequencing and barcoding studies (Bolhaqueiro et al., 2019; Karlsson et al., 2022; Kester et al., 2022). The establishment of organoids from separate tumour regions, possibly combined with the generation of clonal organoid lines at early passage, could prevent the loss of minor subclones (Fujii et al., 2016; Roerink et al., 2018). Evaluating the extent to which genetic heterogeneity is preserved requires sequencing organoids and tumour tissues at sufficient coverage to detect subclonal mutations.

## 5 Conclusion

Lung organoids provide a cell culture platform to study lung development, stem/progenitor cell biology and disease pathogenesis. Several open questions remain, however, concerning their ability to recapitulate the tissue of origin and *in vivo* processes, the ability of organoid collections to accurately reflect population level inter-individual variability and intra-patient heterogeneity (Table 1). We advocate for deeper characterisation of organoids alongside the tissue of origin, for the generation of more diverse organoid biobanks and for greater standardisation of culture media and conditions between laboratories.

**TABLE 1:** 

How closely do lung organoids recapitulate their tissue of origin?	
Challenges	Potential Solutions
• In developmental studies, assessment of cell maturity and identity relies too heavily on expression of markers, which can have low specificity	• Combining marker expression data with spatial information, transcriptomic analysis and organelle characterisation
• Adult lung and cancer organoids lack data to determine to what extent they diverge phenotypically from their tissue of origin	• Proteomic and transcriptomic characterisation of organoids and tissue of origin at the single cell level
	• Systematic identification of the influence of how medium composition and co-culture with non-epithelial (e.g. immune cells) influence organoid phenotype
How well do organoid cultures represent patient cohorts?	
Challenges	Potential Solutions
• Success rates in lung cancer organoid establishment differ widely between laboratories	• Systematic comparison of different lung cancer organoid derivation protocols
• There is poor representation of pre-invasive disease in organoid cancer models	• Identification of transcriptional or genomic features associated with successful lung cancer organoid establishment through collaborative efforts
• Most chronic pulmonary diseases have limited model availability	• Disease modelling using genetic engineering of stem cell derived or non-diseased lung organoids
To what extent does an organoid capture diversity within a patient?	
Challenges	Potential Solutions
• Intra-patient (spatial) heterogeneity is not considered in the establishment chronic pulmonary disease organoids	• More detailed reporting on biopsy location when establishing organoids for chronic pulmonary disease
• There is potential loss of intratumour heterogeneity in lung cancer organoids	• Multi-region organoid establishment combined with high coverage sequencing to detect sub-clonal mutations
